# Global Narratives in Gerontology Education: An Autoethnographic Approach from East Asia

**DOI:** 10.1093/geroni/igaf122.3598

**Published:** 2025-12-31

**Authors:** Takashi Yamashita, Yoon Chung Kim, Giyeon Kim, Tsuann Kuo, Darren Liu

**Affiliations:** University of Maryland, Baltimore County, Baltimore, Maryland, United States; University of Maryland School of Medicine, Baltimore, Maryland, United States; Chung-Ang University, Seoul, Republic of Korea; Chung Shan Medical University, Taichung, Taichung, Taiwan (Republic of China); West Virginia University, Morgantown, West Virginia, United States

## Abstract

Conventional gerontology courses in higher education often prioritize theory and factual content over the cross-cultural experiences of aging, constraining students’ development of cultural competency, such as open-mindedness, empathy, and the ability to challenge age-related stereotypes. While experiential learning, like study abroad, is effective, financial and logistical barriers compromise accessibility for many students. To address this gap, a team of international gerontology educators employed an autoethnographic methodology—a recognized approach for understanding personal and cultural experiences—to conduct immersive fieldwork in Okinawa (Japan), Seoul (South Korea), and Taipei (Taiwan). Through in-depth interactions with local gerontology researchers and community leaders (n = 18), the team collected rich narratives and observations, illuminating unique cultural perspectives on aging. These stories can be integrated into existing gerontology curricula to humanize abstract concepts, highlight cross-cultural differences and commonalities, and challenge age-related stereotypes. For example, a researcher from the Okinawa Centenarian Study shared the historical transition of what it means to be over 100 years old, emphasizing the need to revisit the concept of longevity. Other socioculturally rooted stories, such as a fake bus stop at the care facility in Seoul, and an inclusive support program for diverse older adults with various disabilities, aging stages, and lifestyles at the Red Cross community wellness hub in Taiwan, were illustrated. Such narratives, contextualized with the educators’ lived experiences, can convey insider cultural perspectives in class, promoting their learning and cultural competency. Strategies to effectively share specific narratives with a larger gerontology education community and practical applications are evaluated.

